# Use of prehospital ultrasound in North America: a survey of emergency medical services medical directors

**DOI:** 10.1186/1471-227X-14-6

**Published:** 2014-03-01

**Authors:** John Taylor, Kyle McLaughlin, Andrew McRae, Eddy Lang, Andrew Anton

**Affiliations:** 1 University of Calgary MD program, #108 1990 West 6 Avenue, Vancouver, BC V6J 4V4, Canada; 2 University of British Columbia Department of Emergency Medicine, Vancouver, Canada; 3 University of Calgary Division of Emergency Medicine, Calgary, Canada; 4 Alberta Health Services EMS Calgary zone, Calgary, Canada

**Keywords:** Ultrasonography, Emergency medical services

## Abstract

**Background:**

Advances in ultrasound imaging technology have made it more accessible to prehospital providers. Little is known about how ultrasound is being used in the prehospital environment and we suspect that it is not widely used in North America at this time. We believe that EMS system characteristics such as provider training, system size, population served, and type of transport will be associated with use or non-use of ultrasound. Our study objective was to describe the current use of prehospital ultrasound in North America.

**Methods:**

This study was a cross-sectional survey distributed to EMS directors on the National Association of EMS Physicians (NAEMSP) mailing list. Respondents had the option to complete a paper or electronic survey.

**Results:**

Of the 755 deliverable surveys we received 255 responses from across Canada and the United states for an overall response rate of 30%. Of respondents, 4.1% of EMS systems (95% CI 1.9, 6.3) reported currently using ultrasound and an additional 21.7% (95% CI 17, 26.4) are considering implementing ultrasound. EMS services using ultrasound have a higher proportion of physicians (p < 0.001) as their highest trained prehospital providers when compared to the survey group as a whole. The most commonly cited current and projected applications are Focused Abdominal Sonography for Trauma (FAST) and assessment of pulseless electrical activity (PEA) arrest. The cost of equipment and training are the most significant barriers to implementation of ultrasound. Most medical directors want evidence that prehospital ultrasound improves patient outcomes prior to implementation.

**Conclusions:**

Prehospital ultrasound is infrequently used in North America and there are a number of barriers to its implementation, including costs of equipment and training and limited evidence demonstrating improved outcomes. A research agenda for prehospital ultrasound should focus on patient-important outcomes such as morbidity and mortality. Two commonly used indications that could be a focus of standardized training programs are the FAST exam, and assessment of PEA arrest.

## Background

Ultrasound is a form of medical imaging that is portable, non-invasive and does not expose the patient to ionizing radiation. Healthcare providers that use ultrasound are able to obtain immediate anatomical, diagnostic and functional information on their patients. In recent years, ultrasound machines have decreased in size and cost while producing images of enhanced quality. These advances have made ultrasound more accessible to prehospital care providers.

There is evidence that prehospital ultrasound may be beneficial in diagnosis and management of critically ill patients [1,2] and may be useful in as many as one sixth of medical and trauma EMS missions [3]. EMS providers can be trained to interpret ultrasound scans with a high degree of accuracy in a relatively short period of time [4-6]. For example, prehospital focused abdominal sonography for trauma (FAST) exams have the potential to provide valuable information in abdominal trauma with specificity of 97.5 - 99% and sensitivity of 93 - 100% [2,7] leading to more appropriate transport destination decisions. However, the use of prehospital ultrasound may result in a time delay to hospital by 0–6 min [8]. There is currently insufficient evidence that prehospital ultrasound improves morbidity or mortality in critically ill or injured patients [8,9].

An extensive literature review describes the potential ultrasound indications for prehospital EMS providers [10]. The review concluded that many potential applications exist for prehospital ultrasound but more prospective, outcome-based studies were needed to determine if ultrasound should be implemented more widely. Potential indications for prehospital ultrasound described to date include: FAST [4,11]; echocardiography and assessment of pulseless electrical activity (PEA) arrest [12-14]; cardiac tamponade [15,16]; pneumothorax [17]; assessment of volume status through jugular venous pressure (JVP) or inferior vena cava diameter (IVC) [10]; abdominal aortic aneurysm (AAA) [18,19]; vascular access and intraosseus line placement [20]; endotracheal tube placement [21,22]; fracture identification [23,24]; and identification of pulmonary edema [17,25]. Transmission of ultrasound images to the emergency department has also been described as a possible advantage of prehospital ultrasound [26-28].

While these indications for prehospital ultrasound have been described in the literature, little is known about how EMS services are actually using this technology in the field. We suspect there is significant variation in the adoption of prehospital ultrasound and the perception of indications for its use. We expect that rural services, those with long transport times, and those utilizing air-transport, will be the most common users of prehospital ultrasound. The objective of our study is to describe in detail how ultrasound is currently being used by EMS services in North America.

## Methods

This study was a cross-sectional convenience survey distributed via mail to EMS directors in Canada and the United States. Recipients were identified using the National Association of EMS Physicians (NAEMSP) mailing list and we obtained permission to distribute the survey to NAEMSP members. The University of Calgary Conjoint Health Research Ethics Board approved this study (Ethics ID 24183). The survey consisted of a single mail out and respondents had the option to complete the enclosed paper-based survey or an on-line version of the survey for which the link was provided.

The inclusion criteria for the study were: the survey was to be completed by a medical director of an EMS system, and the EMS system provided prehospital care in Canada or the United States of America.

The main survey consisted of two sections. The first section of the survey consisted of questions that focused on describing the EMS systems of each organization. In the second section, we asked targeted questions based on whether or not the EMS service is using ultrasound.

We calculated proportions of respondents that gave a specific answer to each question and their 95% confidence interval based on a population size of EMS medical directors of 755, the number of NAEMSP medical directors receiving the survey. This number was calculated by subtracting the undeliverable surveys from the total number of surveys sent out. We analyzed each question of the survey independently based on the number of respondents to the question. We compared the characteristics of EMS systems using ultrasound to all responding EMS systems using Fisher’s Exact Test for categorical and binary variables.

## Results

We mailed 766 surveys to EMS medical directors. 11 were returned undeliverable. Of the 755 deliverable surveys, 156 completed the paper-based survey and 69 completed the online survey for a response rate of 30%. Characteristics of the EMS systems are presented in Table 1. Of 222 respondents 9 reported using ultrasound in their EMS service, for an estimated utilization rate of 4.1% (95% CI 1.9, 6.3). Utilization rates were 2.8% (95% CI 0.9, 4.7) and 3.7% (95% CI 0, 7.6) for ground transport and helicopter air transport services respectively. Of 212 respondents that are not using ultrasound, 46 (21.7%, 95% CI 17, 26.4) are considering ultrasound implementation while 166 (78.3%, 95% CI 73.6, 83) are not considering ultrasound implementation. The most commonly cited current and projected applications are FAST and assessment of PEA arrest which are used by 87.5% (95% CI 64.7, 100) of EMS services with ultrasound.

**Table 1:** Characteristics of EMS services

***Characteristic***	***Percent (95% CI)***
**Country** n = 225	
United States	95.1% (92.7, 97.5)
Canada	4.9% (2.5, 7.3)
**Funding** n = 209	
Public	50.7% (44.9, 56.5)
Private	24.4% (19.4, 29.4)
Mixture of public and private	24.9% (19.9, 29.9)
**Urban vs rural population** n = 222	
Urban	30.2% (25.1, 35.3)
Rural	15.3% (11.3, 19.3)
Mixture of urban and rural	54.5% (49, 60)
**Population** n = 222	
0 – 9 999	3.2% (1.2, 5.2)
10 000 – 99 999	23.9% (19.2, 28.6)
100 000 – 999 999	47.3% (41.8, 52.8)
>1 000 000	25.7% (20.9, 30.5)
**Type of transport** n = 222	
Ground transport	95% (92.6, 97.4)
Helicopter air transport	36.5% (31.2, 41.8)
Fixed, wing air transport	8.6% (5.5, 8.6)
**Transport time to nearest hospital** n = 221	
0-10 min	29.4% (24.3, 34.5)
10-20 min	52% (46.5, 57.5)
20-30 min	11.3% (7.8, 14.8)
30-60 min	7.2% (4.3, 10.1)
>60 min	0.0%
**Transport time to nearest tertiary care hospital** n = 221	
0-10 min	13.6% (9.8, 17.4)
10-20 min	38.5% (33.1, 43.9)
20-30 min	19.9% (15.5, 24.3)
30-60 min	16.7% (12.6, 16.7)
>60 min	11.3% (7.8, 14.8)
**Level of training** n = 221	
NREMT Basic or equivalent	3.6% (1.5, 5.7)
NREMT Intermediate or equivalent	1.4% (0.1, 2.9)
NREMT Paramedic or equivalent	67.9% (62.7, 73.1)
NREMT Paramedic with additional training in critical care or registered nurses with critical care training	24.9% (20.1, 29.7)
Physician	2.3% (0.6-4.0)

Among the 8 EMS providers using ultrasound who commented on the level of training of their providers, 4 (50%) are paramedics, 5 (62.5%) are physicians, 1 (12.5%) are rescue medics, and 1 (12.5%) are paramedics or registered nurses with additional training in critical care. Of the 8 EMS services that commented on their ultrasound usage, 7 (87.5%) use it for FAST, 7 (87.5%) assess PEA arrest, 6 (75%) examine for cardiac tamponade, 5 (62.5%) use it to detect AAA, 4 (50%) examine for pneumothorax, 2 (25%) use it for vascular access, 2 (25%) assess volume status through JVP or IVC diameter, and 1 (12.5%) use it to identify fractures. None of the services that responded used ultrasound to confirm intraosseous line placement, endotracheal tube placement, identification of pulmonary edema, or used telemetry of ultrasound images to the emergency department. Among 5 EMS medical directors using ultrasound who commented on the perceived benefits, all 5 (100%) stated that it improves patient management in the field and patient triage, 4 (80%) stated may change disposition upon arrival to definitive care and 3 (60%) stated that it helps expedite care of critically ill patients.

EMS services currently using ultrasound are more likely to have physicians as their highest trained prehospital providers when compared to the survey group as a whole (p < 0.001). Characteristics that were not different between EMS services using ultrasound and the whole survey group included: funding model (public vs private); urban or rural population; size of population base; type of transport (air vs ground); or transport time. All respondents using ultrasound were from the USA, however, this was not a statistically significant association with ultrasound use (p = 0.724) as the majority of survey respondents were American.

The cost of equipment and training are the most significant barriers to implementation of ultrasound with 89.4% (95% CI 85.7-93.1) and 73.7% (95% CI 68.4, 79) of EMS medical directors identifying these barriers respectively. The perceived barriers to implementation of prehospital ultrasound for EMS services that are not using ultrasound are shown in Table 2.

**Table 2:** The barriers EMS Medical directors perceive to implementing prehospital ultrasound n = 198

	**Number of respondents**	**Percentage (95% CI)**
Equipment cost	177	89.4% (85.7-93.1)
Training costs	146	73.7% (68.4-79)
Challenges in training	106	53.5% (47.5-59.5)
Transport times	95	48% (42-54)
Concerns about delaying time to definitive care	90	45.5% (39.5-51.5)
Ultrasound is beyond the scope of practice of providers	76	38.4% (32.6-44.2)
Lack of evidence	76	38.4% (32.6-44.2)
Approval by EMS administration	25	12.6% (8.6-16.6)
Buy-in by other EMS medical directors	21	10.6% (6.9-14.3)
Regulatory factors	29	14.6% (10.4-18.8)

Most EMS medical directors would like data on utility of prehospital ultrasound prior to implementation. 71.8% (95% CI 65.9, 77.7) want studies showing ultrasound improves patient mortality and 73% (95% CI 67.2, 78.8) want studies demonstrating improvements in patient morbidity. Table 3 shows factors and research that would facilitate prehospital ultrasound implementation. In addition to the survey questions, 5 EMS medical directors commented that a cost/benefit analysis would be important research to undertake in the field.

**Table 3:** Factors that would facilitate implementation of prehospital ultrasound among to EMS medical directors not currently using ultrasound

**Research n = 174**	**Number of respondents**	**Percentage (95% CI)**
Studies demonstrating improvement in patient mortality	125	71.8% (65.9-77.7)
Studies demonstrating improvement in patient morbidity	127	73.0% (67.2-78.8)
Studies on sensitivity and specificity of ultrasound used by paramedic operators	97	55.7% (49.2-62.2)
Studies on the effect of prehospital ultrasound on transport time	92	52.9% (46.4-59.4)
Studies about the potential indications for prehospital ultrasound	92	52.9% (46.4-59.4)
Case studies demonstrating examples of prehospital ultrasound and patient outcomes	45	25.9% (20.2-31.6)
**Other facilitating factors n = 165**		
Decreased cost	115	69.7% (63.5-75.9)
Practice guidelines including prehospital ultrasound	109	66.1% (59.7-72.5)
Standardized training available for EMS staff	96	58.2% (51.5-64.9)
Policy statements endorsing ultrasound	74	44.8% (38.1-51.5)

## Discussion

The results of our study indicate that prehospital ultrasound is infrequently used in North America. Prehospital physician providers are associated with increased use of ultrasound. While EMS services commonly use physicians as primary care providers in Europe, in North America physicians have a smaller role in direct patient care and act more often as EMS Medical directors [29]. This difference may explain the low utilization of ultrasound in prehospital care in North America while it may be more feasible in Europe.

Medical directors of EMS systems that are not currently using ultrasound identified a number of barriers to implementation of prehospital ultrasound. The most commonly cited barriers were related to cost of ultrasound equipment and training. Even some EMS services currently using ultrasound identified cost as an ongoing challenge for them.

EMS medical directors identified challenges in training as an important barrier to implementation of ultrasound in their EMS systems. Our study identifies the most commonly used indications for prehospital ultrasound as the FAST exam and assessment of PEA arrest. These indications could be used to create initial prehospital ultrasound curricula. Training for other indications such as AAA screening, vascular access, cardiac tamponade, and pneumothorax imaging could follow a successful template used for FAST and PEA.

Before considering implementing ultrasound into their EMS systems, many directors would like to see additional evidence that prehospital ultrasound improves patient morbidity and mortality. Several EMS directors also specifically mentioned the need for cost-benefit analyses for prehospital ultrasound. The development of a research agenda for prehospital ultrasound could help provide direction for studies that are most likely to change practices. Our data notes that EMS medical directors believe that the objective of a research agenda should be to evaluate impact of prehospital ultrasound on morbidity and mortality.

This study has limitations that arise from using a survey for data collection. The survey is likely to have some degree of selection bias, with respondents that are using ultrasound more likely to respond to the survey because they are already invested in the survey topic (voluntary response bias). Therefore the low levels of use reported are likely are an overestimate of true usage rates and true prehospital ultrasound usage rate could be as low as 1.2% (9/755) if all non-responders represented EMS services not using ultrasound. Respondents were limited to those currently on mailing list of a professional organization, the National Association of EMS Physicians. Although we believe this mailing list is widely inclusive of our target population, it may not be all-inclusive. Our survey response rate of 30% is comparable to other surveys of EMS providers using this survey method [30]. Another limitation arises because not all medical directors completed all sections of the survey. As a result these sections may have some bias in their responses. This phenomenon has previously been reported in surveys of EMS providers [30]. To mitigate this, each question of the survey was analyzed independently based on the number of respondents to that particular question.

## Conclusions

Currently, prehospital ultrasound is infrequently used in North America and EMS services identified a number of barriers to implementation. Current ultrasound use is associated with services with advanced trained providers. Decreased cost for equipment and training may make ultrasound a more feasible expenditure for EMS services. Two commonly used indications that could be a focus of standardized training programs are the FAST exam, and assessment of PEA arrest. A research agenda for prehospital ultrasound may be beneficial and should focus on the impact of prehospital ultrasound on patient outcomes.

## Abbreviations

AAA: Abdominal aortic aneurysm; EMS: Emergency medical services; FAST: Focused abdominal sonography for trauma; IVC: Inferior vena cava; JVP: Jugular venous pressure; NAEMSP: National Association of EMS Physicians; NREMT: National Registry of Emergency Medical Technicians; PEA: Pulseless electrical activity; USA: United States of America.

## Competing interests

The authors have no real or potential conflicts of interest to declare.

## Authors’ contributions

JT developed the research proposal, wrote the ethics application, wrote the grant application, developed the survey, organized and distributed the survey, entered and analyzed data, and was the primary author of final manuscript. KM developed the research question and assisted with the proposal, and he provided expertise in the area of emergency ultrasound. He assisted with ethics application; grant application, survey development and edits of final manuscript. AM assisted with ethics application, grant application, survey design, data analysis and edits of final manuscript and provided expertise in statistical analysis. EL assisted with ethics application; grant application, survey design, data analysis and edits of final manuscript and provided expertise in research methodology. AA assisted with survey design, distributed pilot survey, assisted with edits of final manuscript and provided expertise in prehospital care. All authors read and approved the final manuscript.

## Pre-publication history

The pre-publication history for this paper can be accessed here:

http://www.biomedcentral.com/1471-227X/14/6/prepub
